# Ontogenetic and Among-Individual Variation in Foraging Strategies of Northeast Pacific White Sharks Based on Stable Isotope Analysis

**DOI:** 10.1371/journal.pone.0045068

**Published:** 2012-09-28

**Authors:** Sora L. Kim, M. Tim Tinker, James A. Estes, Paul L. Koch

**Affiliations:** 1 Department of Earth and Planetary Sciences, University of California Santa Cruz, Santa Cruz, California, United States of America; 2 Western Ecological Research Center, United States Geological Survey, Santa Cruz, California, United States of America; 3 Department of Ecology and Evolutionary Biology, University of California Santa Cruz, Santa Cruz, California, United States of America; University of Leeds, United Kingdom

## Abstract

There is growing evidence for individuality in dietary preferences and foraging behaviors within populations of various species. This is especially important for apex predators, since they can potentially have wide dietary niches and a large impact on trophic dynamics within ecosystems. We evaluate the diet of an apex predator, the white shark (*Carcharodon carcharias*), by measuring the stable carbon and nitrogen isotope composition of vertebral growth bands to create lifetime records for 15 individuals from California. Isotopic variations in white shark diets can reflect within-region differences among prey (most importantly related to trophic level), as well as differences in baseline values among the regions in which sharks forage, and both prey and habitat preferences may shift with age. The magnitude of isotopic variation among sharks in our study (>5‰ for both elements) is too great to be explained solely by geographic differences, and so must reflect differences in prey choice that may vary with sex, size, age and location. Ontogenetic patterns in δ^15^N values vary considerably among individuals, and one third of the population fit each of these descriptions: 1) δ^15^N values increased throughout life, 2) δ^15^N values increased to a plateau at ∼5 years of age, and 3) δ^15^N values remained roughly constant values throughout life. Isotopic data for the population span more than one trophic level, and we offer a qualitative evaluation of diet using shark-specific collagen discrimination factors estimated from a 3+ year captive feeding experiment (Δ^13^C_shark-diet_ and Δ^15^N_shark-diet_ equal 4.2‰ and 2.5‰, respectively). We assess the degree of individuality with a proportional similarity index that distinguishes specialists and generalists. The isotopic variance is partitioned among differences between-individual (48%), within-individuals (40%), and by calendar year of sub-adulthood (12%). Our data reveal substantial ontogenetic and individual dietary variation within a white shark population.

## Introduction

Although diet is often treated as a species-level trait, variation in diet composition and foraging behavior occurs within most species. This variation can be attributed to at least three factors–habitat-specific variation in prey availability; differences in the cost-benefit ratios of potential prey among the sexes, or age- or size-classes of consumers; and phenotypic variation among what often appear to be otherwise similar individuals [1]–[5]. Collectively, this dietary variation influences the fitness of consumers and their ecological and evolutionary impacts on prey species, communities, and ecosystems [1]–[5].

White sharks (*Carcharodon carcharias*) are apex predators that can have cascading effects on marine ecosystems [6], [7], but our understanding of their foraging ecology is fragmentary and often biased by spectacular accounts, especially attacks on humans and other large mammals. In the northeastern Pacific Ocean, white sharks were once considered a nearshore species that preyed primarily on pinnipeds, a perception arising from many studies focused on coastal sites near pinniped colonies where shark foraging behavior was easy to observe [8]–[13]. This view has been challenged by recent satellite tagging data from white sharks off the coast of California and Baja California, Mexico, which revealed migration between the North American continental shelf and two offshore areas (18 to 26°N and 125 to 140°W) [14]–[17]. Isotopic data from tagged individuals corroborated offshore foraging on lower trophic level prey and indicated similar dietary preferences within this population [18]. Although observations of white shark predation on non-pinniped prey are rare, stomach contents include remains from invertebrates, turtles, fish, and sharks [19]. Here, we assess population-level diet variation, potential ontogenetic shifts in prey preferences, and individual diet specialization through analysis of carbon and nitrogen isotope variation.

The stable isotope composition of a tissue reflects a temporal integration of dietary and environmental inputs (albeit mediated by animal physiology), and can thus be used as a natural tracer for foraging variation. The most commonly used stable isotope ratios in dietary studies are ^13^C/^12^C and ^15^N/^14^N. Physiological processes lead to a subtle increase in ^13^C- and ^15^N-concentration with trophic level in consumers (∼1‰ and ∼3‰ per trophic step, respectively) [20]. In addition, there are ^13^C/^12^C and ^15^N/^14^N gradients throughout the eastern basin of the Pacific Ocean controlled by a variety of factors related to primary production at the base of the food web [21]–[23]. For carbon, upwelling brings ^13^C-depleted deep water to the surface and nutrient-driven phytoplankton blooms off the California coast increase the baseline carbon isotope values ∼2–3‰ relative to less productive offshore areas [21]–[23]. The spatial nitrogen isotope gradient has a similar range, but is more complex as the source of nutrients (i.e., nitrate, ammonium, N_2_) and processes of biological incorporation or geochemical cycling (i.e., N_2_ fixation, nitrate or ammonium assimilation, or denitrification) vary with productivity regimes [23]–[27]. These baseline trends are conserved in consumer tissues; for example, pinnipeds foraging in productive ^13^C-enriched nearshore habitats on the Pacific Rim have carbon isotope values ∼2‰ greater than those foraging offshore [28].

Assessments of consumer diet with stable isotope analysis must address these spatial and trophic variations among potential prey. This technique has been used to chronicle patterns of diet and habitat use in a variety of marine predators, including marine mammals (see review by [29]), marine turtles [30]–[32], and sharks [33]. Two issues that are important to disentangling the effects of spatial versus trophic variations on consumer isotope values are biological parameters (i.e., discrimination factors and turnover rates) and distinct prey isotope values. In complex systems, however, these sources of variation are difficult to tease apart. The few isotopic studies featuring sharks often calculate trophic position and report isotopic differences among tissues [33]–[39], but other ecological insights, such as dietary preferences and habitat use, are limited.

The concept of ecological niche [40] was adopted in isotopic ecology because stable isotopes vary with aspects of a consumer’s diet and habitat through time [41]. Although ecological and isotopic niches cannot be directly translated, isotopic variation within and among individual consumers of a particular species or population reflects the niche width [41]–[43]. Data from multiple individuals can yield an estimate of population-level isotopic niche width, but to capture within-individual dietary variation, multiple measurements of an individual’s diet and habitat preferences over time are required. Serially-sampled accretionary structures (i.e., feathers, baleen, vibrissae, turtle scutes, shark vertebrae, etc.) produce ontogenetic time series (e.g., [32], [36], [43]–[46]) and can be used to establish within-individual isotopic niche width [32], [36], [43]–[46]. These patterns can be compared among individuals to identify generalists with overlapping isotopic values or specialists that occupy just part of the population’s isotopic range [43]. Although bony fish are featured in studies of individuality, we are aware of only one other study featuring elasmobranchs (shark, skates, and rays) despite their considerable diversity, wide distribution, and functional importance in marine and estuarine systems [2], [33].

We investigated isotopic time series from the vertebrae of white sharks collected from the northeast Pacific Ocean between 1957 and 2000. Because white shark vertebrae grow by accretion, isotopic values from growth bands record lifetime variations in an individual’s diet. We hypothesized δ^15^N values to increase with age to reflect a shift to high trophic-level prey. Furthermore, a quantitative analysis of carbon and nitrogen isotope data will reveal the degree of dietary variation within and among individuals in this white shark population.

## Methods

### (a) Collection and Preparation of Vertebrae for Isotopic Analysis

The vertebrae of 15 adult white sharks were sampled from existing collections (Table 1). Fourteen specimens were caught off the California coast (locations are listed in Table 1, if available). One specimen (42094–2) was caught offshore from Baja California, Mexico, and may be part of the population that aggregates near Guadalupe Island [17]. Because the isotopic pattern from this individual was similar to that for some of the sharks caught off the California coast, we included data from this shark in our analyses.

**Table 1:** Summary of biological and collection data and proportional similarity index (*w_ij_*) for white sharks.

ID	Year of death	Sex	# Growth bands	Location caught	Collection of acquisition	Mean adult		*w_ij_*	Method of storage
						δ^13^C value,‰ (SD)	δ^15^N value,‰ (SD)		
26245	1957	**F**	20	Monterey Bay, CA	CAS	−13.3 (0.6)	20.2 (0.3)	0.17	Dry
26678	1959	?	11	Selva Beach, CA	CAS	−11.4 (0.2)	18.8 (1.0)	0.23	Dry
27015	1960	?	9	Stinson Beach, CA	CAS	−12.0 (0.2)	18.1 (0.4)	0.25	Ethanol
26781	1960	?	9	Tomales Bay, CA	CAS	−11.6 (0.3)	18.9 (0.5)	0.24	Ethanol
42094–2	1965	?	7	Eastern Pacific, Baja Mexico	LACM	−14.1 (0.6)	16.7 (0.3)	0.33	Dry
WS CM	1976	?	10	Point Reyes, CA	GC	−13.6 (0.6)	17.6 (0.7)	0.50	Dry
WH 17	1980	?	14	California	MLML	−13.8 (0.8)	19.0 (0.6)	0.73	Frozen
WS 21	1983	M	16	Anacapa Island, CA	MLML	−14.9 (0.2)	16.0 (0.4)	0.26	Frozen
WS 100	1985	?	19	Santa Barbara, CA	MLML	−12.6 (0.6)	18.1 (0.8)	0.90	Frozen
WS KG	1986	?	13	SE Farallones, CA	KG	−13.0 (0.5)	19.6 (0.2)	0.59	Dry
WS 101	1991	?	11	CA	MLML	−13.4 (0.2)	16.6 (0.4)	0.74	Frozen
WS 128	1992	?	17	CA	MLML	−13.3 (0.8)	18.4 (0.9)	0.42	Frozen
42898 PR	1998	?	15	Point Reyes, CA	SA	−12.4 (0.5)	18.5 (0.3)	0.08	Dry
56731–1	2000	?	17	Catalina Island, CA	LACM	−12.3 (0.5)	19.0 (0.8)	0.73	Dry
CC3	2000	F	14	Morro Bay, CA	LML	−12.0 (0.2)	19.8 (0.5)	0.33	Frozen

Abbreviations are as follows: California Academy of Sciences (CAS), Natural History Museum of Los Angeles County (LACM), G. Chan (GC), Moss Landing Marine Lab (MLML), K. Goldman (KG), S. Anderson (SA), and Long Marine Lab (LML).

All of the vertebrae were stored frozen or dry, with two exceptions (27015 and 27681), which were preserved in alcohol until sampling (Table 1). While some prior studies have found greater isotopic variability and a shift to lower C:N_atomic_ values for ethanol-preserved muscle [47], [48], we found no differences in isotopic or C:N_atomic_ variability between frozen and ethanol preserved vertebrae, and so we included data from ethanol preserved specimens in our analyses.

White shark vertebral centra grow by accretion in concentric rings and have annual growth bands that are used to age individuals [49]. We followed sampling and preparation techniques in Kim and Koch [48] for these vertebrae. Briefly, a 1-cm thick section was cut from the parasagittal plane with a diamond saw. Then, sections were polished and annual rings, defined as one opaque and one translucent growth band pair, were independently counted and marked by SLK and a researcher who conducts elasmobranch age and growth studies at California State University, Moss Landing Marine Lab (MLML). Individual growth rings were sampled from the corpus calcerum using a New Wave MicroMill (MLML and the Marine Analytical Lab, University of California, Santa Cruz [UCSC]) from a maximum depth of 1.2 mm (File S1). Samples were decalcified using 0.5 M EDTA (pH 8), rinsed 10 times with milliQ water to isolate collagen, and freeze-dried for stable isotope analysis (method modified from [48]). Samples were weighed to 300–400 µg into tin boats (3×5 mm, Costech) and analyzed at the Stable Isotope Laboratory at UCSC on an elemental analyzer coupled to an isotope ratio-monitoring mass spectrometer (Thermo-Scientific Delta-XP IR-MS). Isotope ratios are expressed in δ values, where.
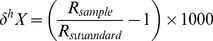
(1)


X is the element of interest, h is the high mass number, and R is the high mass-to-low mass ratio. Units are parts per thousand (per mil, ‰) deviations from a standard. The δ^13^C and δ^15^N values were referenced to V-PDB and AIR, respectively. Replicates of a gelatin standard within each analysis session allowed for mass and drift corrections. Comparisons of this standard within and between runs yielded SD of <0.2‰ and <0.3‰ for δ^13^C and δ^15^N values (n = 77), respectively.

Because of the temporal span of our data, we corrected for anthropogenic changes in the δ^13^C values of Earth surface carbon reservoirs that occurred over the past 70 years [50]. As δ^13^C values did not change constantly through this period, two atmosphere-derived rates (0.05‰ per year for 1937–1960 and 0.022‰ per year for 1961–2000) were used to correct samples to 2000, the year of death for the most recent individuals [51].

### (b) Quantitative Analysis of Ontogeny

To test for the existence of an ontogenetic shift at the population level in a generalized linear model, we established 2 age classes and had individuals as a source of variation to determine if δ^15^N values varied significantly with age using a one-way analysis of variance (ANOVA). These age classes were based on length data from observation and tagging studies of white sharks near pinniped rookeries. The smaller white sharks observed at Año Neuvo Island, Southeast Farallon Islands, Tomales Bay, and Point Reyes are approximately 300–350 cm long [16], [52]. Additionally, white shark teeth develop finer serrations beginning at 300 cm, indicating a functional shift [53]. Age and growth studies often use annual vertebral growth bands [49] and relate them to length or mass based on von Bertalanffy growth functions (VBGF, [54]):
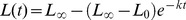
(2)where t is time, L_∞_ is the maximum (or asymptotic) length, L_0_ is length at birth (t = 0), and k is an empirically derived growth constant. According to VBGF, 300 cm corresponds to white sharks that are 5 years old [55], [56]. Based on tooth morphology and white shark presence at pinniped rookeries, we classified isotopic data from growth bands corresponding to birth through the end of the 5^th^ year as “young” and to ≥6 years old as “sub-adult to adult.”

We next evaluated individual differences in ontogenetic dietary variation. For each shark, we fit three alternative functions to the time series data: i) a constant δ^15^N value (assuming random variation but no prevailing trend with age); ii) a continuous increase in δ^15^N values with age, fit using a first-order polynomial; and iii) a non-linear, asymptotic increase in δ^15^N values with age, fit using VBGF (Equation 2, substituting δ^15^N values for length). We removed statistically significant outlier data (studentized residuals that fall outside 95% prediction interval for new data points) prior to final fitting. We tested the statistical significance of each function at á = 0.05, and used degrees-of-freedom-adjusted R^2^ values to evaluate goodness of fit for each model. We then assigned individuals to one of three classes based on which of these models (no ontogenetic shift, linear increase or asymptotic increase) was significant and provided the best fit. For individuals exhibiting an asymptotic increase, we designated the age of transition from young to sub-adult through adult diets as the point at which the VBGF function equaled 90% of the asymptotic value. For individuals exhibiting no significant ontogenetic shift and having high δ^15^N values in early growth bands, we sampled and analyzed portions of the central vertebra, which form from maternal resources before parturition, to assess the potential of metabolic turnover within the vertebra.

### (c) Potential Prey for Qualitative Assessment of White Shark Diet

We compiled isotopic data for potential prey gleaned from studies in northeastern Pacific ecosystems to provide a dietary context for our sub-adult to adult isotopic data from white sharks. We limited our assessment to qualitative patterns, rather than estimating specific proportions of dietary inputs, because the large number of potential prey and their variance would likely produce indeterminate results [57]. The potential prey we included were as follows: California sea lion [28], [40], [58]; harbor seal [28], [41]; northern elephant seal [28], [41]–[43]; harbor porpoise [32], [36], [43]–[46], [59]; dolphins (Berman and Newsome, unpublished data); various tuna species [32], [36], [43]–[46], [60]; neritic fish [43], [61]; blue and hammerhead sharks [62]; and cephalopod species [63], [64]. The localities and isotopic values for all prey are compiled in File S2. All prey isotope values were “corrected” (and error propagated) to resemble collagen for comparison.

While our shark data span from 1950–2000, the prey data are relatively recent and thus may not reflect secular shifts in baseline isotope values due to climate change or changes in productivity. This source of variation is difficult to constrain, but there is little evidence for major secular shifts (i.e., >1 or 2‰), and variation of this magnitude will not affect our qualitative assessment of diet.

### (d) Assessing the Degree of Individuality

To evaluate niche overlap, we used Pianka’s measure [65] under multivariate normality [66], which is as follows:
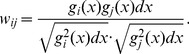
(3)


Equation 3 allows for the calculation of proportional overlap (in 2-dimensional niche space) between two individuals or populations (*i* and *j*), accounting for multivariate covariance and density distributions [*g(x)*]. We measured the degree of isotopic niche overlap, or “proportional similarity,” between each individual’s sub-adult/adult isotopic range and the population-level isotopic range (i.e., all sub-adult/adult growth bands for 15 individuals). Averaged across individuals, this proportional similarity index (*w_ij_*, Equation 3; [66]) allowed us to examine the degree of individual specialization; individual specialists would be expected to have a low degree of overlap (*w_ij_* <<1), while generalists would have extensive overlap (*w_ij_* ≈ 1).

We also assessed the effects of individual differences on sub-adult/adult isotopic values within a generalized linear model. This analysis allowed us to assess the relative amount of variance explained by differences among individuals vs. variation within individuals. We included a temporal category, “calendar year of sub-adulthood,” defined as the calendar year a shark reached 6 years of age. The three categories for calendar year of sub-adulthood were based on pinniped populations: before the passage of the Marine Mammal Protection Act (pre-1972), during the period when pinniped populations were increasing (1972–1986), and after pinniped populations doubled from pre-1972 counts (post-1986) [67], [68]. Other biological details (i.e., sex, location caught, etc.) were not included in our analysis because the information was not available for all specimens (Table 1). The best-fit models for δ^13^C and δ^15^N values (weighted equally and independently) were selected based on minimal Akaike Information Criterion (AIC) values [69]. All statistical analysis was performed in MatLab (version 8.0).

### (e) Discrimination Factors

There are offsets between prey and consumer δ^13^C and δ^15^N values, known as trophic discrimination factors, which reflect preferential sorting during metabolism and incorporation into tissues [20], [70], [71]. To compare potential prey and consumer isotope values, discrimination factors defined as:
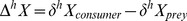
(4)must be applied to account for trophic enrichment of ^13^C and ^15^N. The average carbon and nitrogen discrimination factors that are widely used are 0.4‰ (SD = 1.3‰) and 3.4‰ (SD = 1.0‰), respectively [72], but actual values vary with diet, physiology, and tissue [22], [73], [74].

We conducted a controlled feeding study with captive leopard sharks (*Triakis semifasciata*) fed squid over 1250 days [62]. The care and protocol for euthanizing the leopard sharks were approved by the UCSC Chancellor’s Animal Research Committee (permit code: Koch 0901) and were in accordance with Institutional Animal Care and Use Committee (IACUC) standards. Briefly, the leopard sharks (n = 3) were caught in the San Francisco Bay from August 2005 to January 2006 and maintained at Long Marine Lab, UCSC in polyethylene tanks (2.3 m diameter, 1.2 m water depth) with a continuous flow of filtered seawater from the Monterey Bay (temperature range: 13°–17°C; salinity range: 30–34) until July 2009. The sharks were sacrificed using a lethal dose of tricaine methanesulfonate (MS-222) and vertebrae were extracted and frozen at −20°C. A pair of adjacent vertebrae from the anterior column were cleaned and selected for analysis. For each pair of vertebrae, one vertebra was thin-sectioned to measure growth bands (following sectioning methods of [75] and adapted by [76]) and growth bands in the other vertebra were drilled and collagen prepared for stable isotope analysis. Growth bands were measured 3 times from each shark’s vertebrae non-consecutively using a microscope and transmitted light. The outermost bands without statistically different isotopic values were averaged as the δ^h^X_consumer_ value in Equation (4).

## Results

### (a) White Sharks

**Figure 1:**
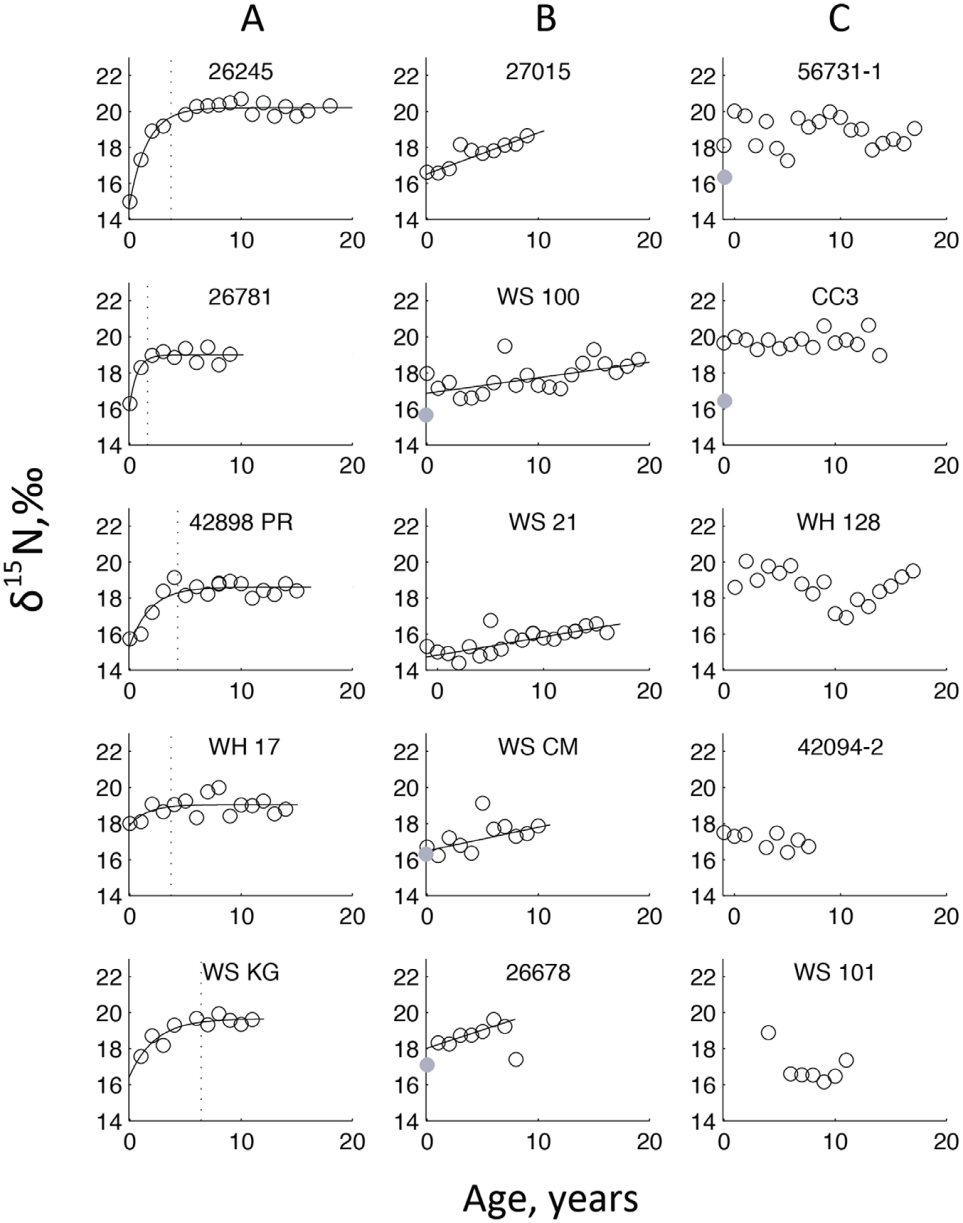
δ^15^N values versus growth increment number (age) for 15 white sharks. A) Individuals modeled with a VBGF curve. B) Individuals showing a significant linearly increasing trend. C) Individuals showing no significant pattern. Average pre-parturition δ^15^N values (n = 3) are indicated as grey filled circles for individuals with relatively high juvenile δ^15^N values (>17‰).

A comparison of young and sub-adult to adult white sharks, blocked across individuals, demonstrated a significant ontogenetic shift (F_1, 206_ = 23.19, p<0.0001, r^2^ = 0.69), confirming that there is an ontogenetic shift in dietary preferences or habitat use in the northeastern Pacific white shark population. Five individuals showed a non-linear, asymptotic increase in δ^15^N values, with the transition to the sub-adult to adult diet occurring at approximately 4 years of age (Figure 1A). Five individuals exhibited a linear relationship between δ^15^N and age, with a mean increase of 0.127 yr^−1^±0.073 (p<0.0001; Figure 1B). The remaining 5 individuals showed no significant relationship between age and δ^15^N values (Figure 1C). For individuals with high δ^15^N values (>17.0‰) before age 6 and linearly increasing or no ontogenetic shift (i.e., 26678, 56731–1, CC3, WS 100, WS CM), the average δ^15^N values in the central vertebra, which are formed prior to parturition, were 0.6–3.4‰ less than the growth bands for ages 1–5 (Figure 1). A comparison of individual age vs. δ^13^C values did not reveal significant patterns (File S3). These three patterns of individual variation in δ^15^N values within the population are robust and point to substantial differences in the ontogeny of foraging behavior among individuals.

**Figure 2:**
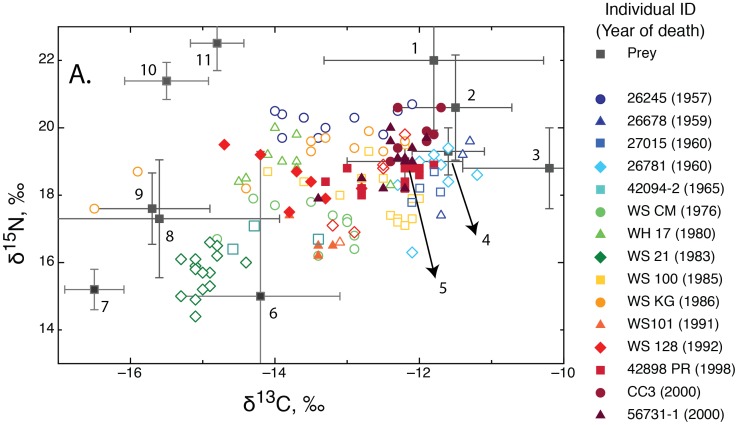
Carbon and nitrogen isotope values from sub-adult to adult growth bands (≥6 years old). The colored symbols are from white sharks; open symbols represent years ≤1986 and closed symbols represent years >1986. Isotopic values for potential prey data are the grey boxes and are as follows: 1) northern elephant seal, 2) California sea lion, 3) harbor seal, 4) dolphin, 5) harbor porpoise, 6) tuna, 7) neritic fish, 8) offshore cephalopod, 9) nearshore cephalopod, 10) blue shark, 11) hammerhead shark. The mean prey isotope values were corrected for trophic enrichment (Δ^13^C = 4.2‰ and Δ^15^N = 2.5‰) and collagen-to-muscle (Δ^13^C = 2.0‰ and Δ^15^N = 0‰), if necessary (prey data and citations are listed in File S2).

A bivariate plot of isotopic data from sub-adult/adult growth bands illustrates the dietary diversity within the northeast Pacific white shark population (Figure 2). The population-level δ^13^C and δ^15^N values range from –14.5 to –11‰ and 17 to 21‰, respectively. Within this range, certain individuals (e.g., WS CM, WS 100, WS 128) exhibit isotopic ratios consistent with a diet rich in lower trophic level prey, whereas other individuals (e.g., 26245, WS KG, CC3) appear to consume primarily high trophic level prey. One individual, WS 21, was an outlier from the population pattern, with low δ^13^C and δ^15^N values.

**Figure 3:**
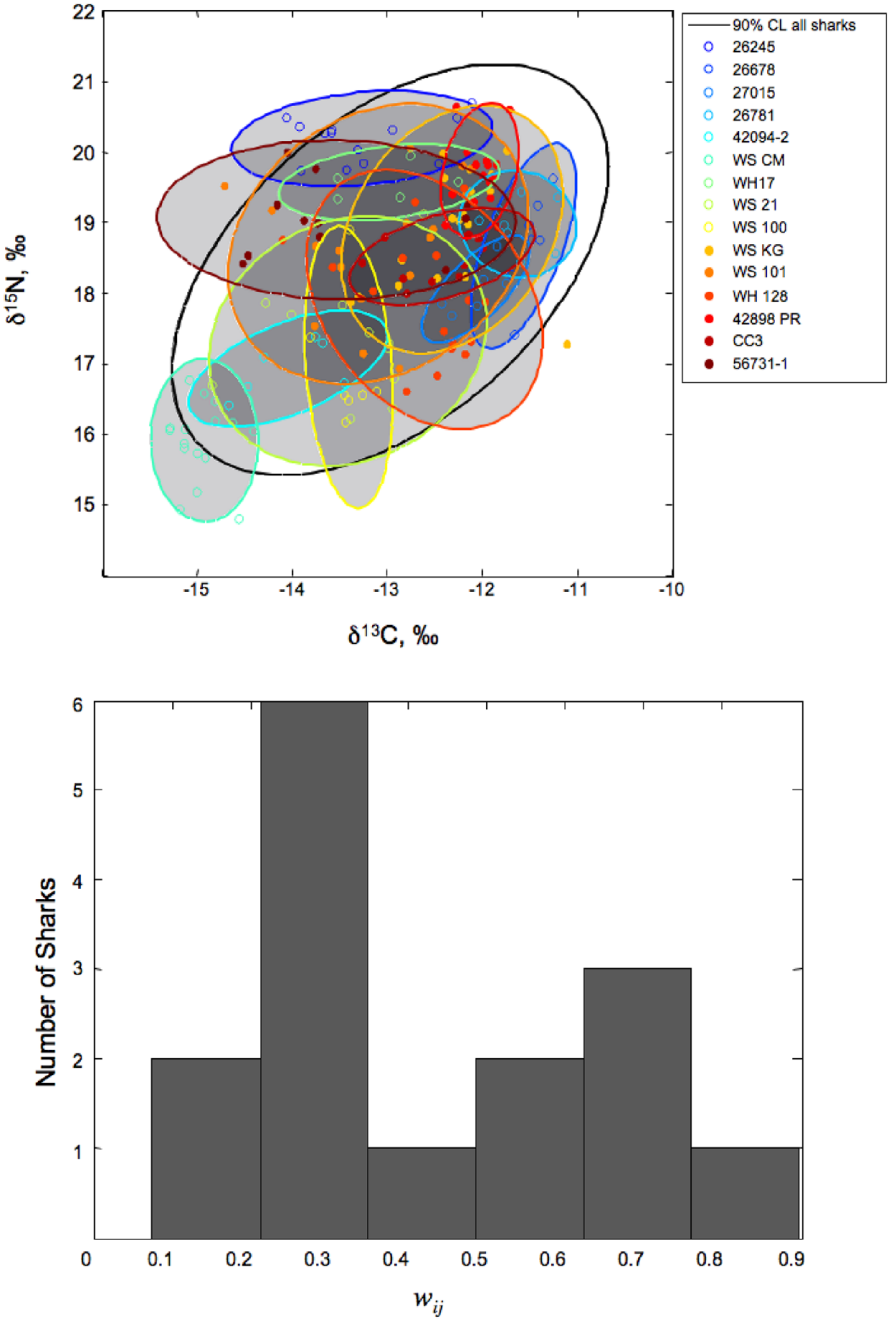
The niche overlap between each individual and the population. A) The 90% confidence limit for the population (black ellipse) and for individual sharks (colored ellipses). B) The distribution of the proportional similarity index, *w_ij_* [66], within the sampled population of California white sharks, which exhibits strong individuality with both specialists and generalists.

Our data reveal that northeast Pacific white sharks occupy a wide isotopic niche, as expected for a generalist population. However, closer inspection reveals a range of strategies among individuals, which may relate to sex, location, size, or individuality, as illustrated by the size and placement of individual bivariate confidence ellipses relative to the population (Figure 3A). The sharks in the sample population varied considerably in terms of isotopic overlap, with most sharks having low degrees of overlap (*w_ij_*<0.5), but a few having high values (*w_ij_*<0.8) suggesting more generalized diets (Figure 3B). The modal range of the *w_ij_* value was 0.23–0.33 for this population (Table 1). The generalized linear model of δ^13^C and δ^15^N data showed significant effects of individual variation (F_12,192_ = 22.76, p<0.0001). The combined variance in δ^13^C and δ^15^N values was explained largely by differences among individuals (48%) and within-individual effects (40%). The calendar year of sub-adulthood accounted for 12% of the variance in a model with both isotope values weighted equally. Post hoc comparisons using the Tukey HSD test indicated the significance of all pairwise differences between calendar year of sub-adulthood categories for both isotopes (all p-values <0.0001 except between pre-1972 and post-1986 δ^13^C means [p = 0.018]).

### (b) Discrimination Factors for Vertebrae

Because dietary carbon and nitrogen incorporate relatively slowly into shark tissues [77], discrimination factors were based on the average isotopic values from the last 1–1.5 years of the experimental sharks (outermost 12 mm). The average width for the last 6 bands (representing the final 3 years for the shark) differed among individuals, but the total thickness for the final 3 years ranged from 15.3 to 18.6 mm (Table 2). Average δ^13^C and δ^15^N values from the outermost 12 mm did not differ significantly among sharks (Kruskal-Wallis Test, δ^13^C values: H = 3.51, 2 d.f., p = 0.17 and δ^15^N values: H = 1.32, 2 d.f., p = 0.52; Table 2). The average δ^13^C and δ^15^N values (n = 6, SD) near the birthmark, where the corpus calcerum changes angle, was −15.4‰ (0.3) and 18.5‰ (0.5), respectively, which is significantly different from the outermost bands that represent body tissues in steady state with the captive squid diet (Kruskal-Wallis Test, H = 12.5, 1 d.f., p = 0.0004). Based on the average isotopic value of the sharks’ diet (Table 2; [62]), the vertebral collagen Δ^13^C and Δ^15^N values (SD) are 4.2‰ (0.7) and 2.5‰ (1.1), respectively.

**Table 2:** Average width of last 6 growth bands and average isotopic values from outer-12 mm of vertebrae from leopard sharks fed a constant diet of squid over 1250 days.

Individual	Average width of last 6 growth bands ± SD (n), mm	Average δ^13^C value ± SD (n), ‰	Average δ^15^N value ± SD (n), ‰
Diet		−18.5±0.3 (43)	13.3±0.7 (43)
CS	3.10±0.38 (18)	−14.1±0.4 (8)	15.9±0.8 (8)
FS	2.54±0.51 (18)	−14.4±0.5 (8)	16.1±1.1 (8)
FL	2.99±0.41 (24)	−14.6±0.3 (4)	15.3±0.5 (4)

## Discussion

Isotopic analysis of white shark dietary patterns reveals ontogenetic and among-individual variation. This finding contrasts with previous dietary assessments based on coastal observations and stable isotope data, which suggest that nearshore pinnipeds were the preferred prey for this population [18], [19], [35], [46].

### (a) Ontogenetic Patterns

Previous studies [16], [36], [52], [53] have suggested a shift from low to high trophic level prey with age in white sharks, and the overall increase in δ^15^N values with age reported here is consistent with this scenario. We expected the time series from all sharks to exhibit a trend of early increase in δ^15^N values, followed by a plateau once individuals had switched to a high-trophic level adult diet. While this pattern was evident for some individuals, it was not the dominant trend in our sample (Figure 1). The variation in ontogenetic patterns cannot be explained by long-term environmental changes, as sharks that exhibited the asymptotic increase in δ^15^N values and those that did not spanned the temporal range of our study.

The lack of a rise in δ^15^N values in some individuals is due to high values in years 1–5. Below, we discuss three possible explanations for these high values.

1. If young sharks scavenged carcasses of pinnipeds or large squid [78], they would have high δ^15^N values. Because feeding observations of juvenile white sharks are rare, this hypothesis is untested.
2. A residual signal from maternally-derived nutrients may label these early growth increments because of long incorporation rates [77]. We consider this unlikely, as rapid juvenile growth [56], [79] likely erases the isotopic signal from maternal resources beyond the first growth increment.
3. A small but significant amount of metabolic turnover within vertebral centra could label growth bands 1–5 with material that reflects the high trophic level diets of adults. This explanation would require near complete turnover of collagen in grown increments 1–5, which seem unlikely given the densely mineralized acellular cartilage in shark vertebrae, but remodeling could occur during sustained swimming, as evidenced in bony fish [80], [81].

### (b) Vertebrae Discrimination Factors

The trophic enrichment used for white shark prey comparisons was based on the leopard shark discrimination factors (Δ^13^C and Δ^15^N values equal 4.2‰ and 2.5‰, respectively). These shark-specific collagen discrimination factors are greater than other tissues (i.e., blood or muscle) [22], but similar to other collagen discrimination factors [82], [83]. Because collagen has a high glycine content, which is relatively ^13^C-enriched compared to other amino acids [84], its δ^13^C values are greater than muscle. In contrast to our study, previously published shark vertebrae discrimination factors were obtained from relatively short growth periods (<200 days) and collagen was not isolated within the calcified vertebral tissue [85], [86]. Because organic components within a tissue can have different isotopic values and vary between individuals and species, it is important to isolate and compare similar substrates, when possible. Furthermore, the prey isotope values of these previous studies are confounding factors, as Hussey et al. [85] estimated prey values from weight-based feeding logs and Malpica-Cruz et al. [86] fed sharks a pellet diet that was low in protein relative to natural diets, two important factors in determining discrimination factors [74], [87], [88].

### (c) Assessment of Sub-adult to Adult White Shark Diet

Isotopic results from white shark vertebrae indicate a diverse diet and support their classification as a generalist population (Figure 2). Representing the entire dietary range of white shark prey species and localities is not feasible. Therefore we present data on a subset of common potential prey identified from stomach content studies that span a diverse range of trophic levels and habitats (Figure 2). One potential prey group we omitted was large whales because white sharks selectively consume their blubber [11] and collagen is primarily routed from dietary protein [89], [90].

Although most white sharks exhibit a range of intermediate isotopic values, which is consistent with previous isotopic evidence for both nearshore and offshore foraging [18], strategies may vary among individuals. In combination, isotopic values, ontogenetic patterns and *w_ij_* values can indicate the extent of pinniped consumption. For example, individuals with intermediate isotope values, ontogenetic shift to higher trophic level, and a high degree of specialization (27015, 26781, and WS KG) are likely foraging on pinnipeds when near shore. However, intermediate isotopic values suggest that there are some offshore inputs (with lower δ^3^C and δ^15^N values). Other individuals (i.e., WH 17, WS 100, WS 128, 56731–1) that also have intermediate isotope values, but with little to no ontogenetic shift and a low degree of specialization, are likely opportunistic, non-specialized foragers. One distinct outlier among the sharks in our population is WS 21, which had low δ^13^C and δ^15^N values throughout its lifetime (Figure 2). These isotopic values suggest this individual did not consume marine mammals and its foraging ecology likely diverged from the well-studied California and Baja populations [14]–[18].

These isotopic results demonstrate the broad dietary range of white sharks, but caution should be taken when attempting to determine prey more specifically. The prey isotope values (after trophic enrichment correction) mostly encompass the white shark data, but overlap of a consumer’s δ^13^C and δ^15^N values with a prey could also result from integration across several outlying prey taxa. For example, the isotopic values for 26781 overlap with dolphin and harbor porpoise (preys 4 and 5, respectively, in Figure 2) whereas CC3’s values overlap with California sea lion, dolphin, and harbor porpoise (preys 2, 4, and 5, respectively, in Figure 2). However, it is likely that the isotopic mixing space for these sharks also included the following outlying prey: northern elephant seals, harbor seals, tuna, off-, and nearshore cephalopods (preys 1, 2, 6, 8, and 9 respectively, in Figure 2). Overall, the isotopic data for 26781 and CC3 suggest marine mammals were the dominant prey but cephalopods and tuna were also likely consumed when individuals were offshore, similar to results from satellite tagged white sharks [18].

### (d) Individuality

The isotopic data in aggregate suggest that northeast Pacific white sharks are generalists at the population level, but further analysis reveals a high degree of individual specialization within the population. Individual differences within the white shark population can be attributed to changes in prey preference and foraging location with ontogeny. A core constraint on our analysis of niche occupancy and breadth is that if isotopic values differ between two specimens, then either prey type or foraging location (or both) must differ between the specimens. The converse is not true, however. Isotopic similarity between two specimens could result from consumption of the same prey in the same location, but also from fortuitous combinations of different prey types in different locations. Because we do not attempt to specify the particular prey, our assessment of individuality is a conservative measure and reflects minimal niche differences.

The proportional similarity index (*w_ij_*) quantified and compared isotopic variation within each individual to the population’s isotopic distribution. The *w_ij_* values for the sampled population indicated 8 specialists (*w_ij_* = 0–0.33), 4 generalists (*w_ij_* = 0.73–0.90), and 3 animals with intermediate values (*w_ij_* = 0.42–0.59; Table 1). We note the distinction between *w_ij_* (which describes the proportional overlap between an individual’s isotopic values and the population’s average) and isotopic niche breadth (the absolute range of isotopic variation in an individual), as these two metrics are not necessarily correlated. For example, isotopic niche breadth was similar for specimens 42094–2 and 42898 PR (represented by individual isotopic distributions in Figure 3A), but their *w_ij_* values were 0.33 and 0.51, respectively (Table 1). The higher *w_ij_* value for 42898 PR reflects the fact that its isotopic niche, while narrow, overlaps with a greater number of conspecifics.

### Conclusions

Stable isotope analysis of white shark vertebrae provided lifetime records of diet and revealed a variety of feeding patterns. For example, we found significant variability in the degree and timing of shifts in δ^15^N values with age among individuals. Although there was a significant difference between young and adult diets, not all individuals displayed a shift to a higher trophic level prey. The isotopic data suggest that as a population, sub-adult to adult white sharks are generalist predators and consume a diverse array of high and low trophic level prey from nearshore and offshore habitats. However, a comparison between individual and population isotopic niche overlap revealed a high degree of dietary individuality.

Although confounding factors, such as the lack of biological metadata and unknown effects of physiological characteristics, prevent specific dietary assessments, this study provides the first evidence of ontogenetic and individual dietary variation in white sharks. One potential explanation for this pattern may be a mechanism to increase foraging success in species experiencing high intraspecific competition (i.e., the niche variation hypothesis [91]). Instead of individuals using all resources (i.e., potential prey or foraging locations) equally, individual foraging patterns diverge to create a spectrum of specialists that do not differ in survival or reproductive success [2], [4], [43], [92], [93]. This isotopic study, in conjunction with satellite tagging studies, is expanding our understanding of the foraging ecology of white sharks.

## Supporting Information

File S1 Photo showing annual growth bands before (left) and after drilling (right) on specimen WS101. Ages are also noted next to the growth bands.
(TIF)

File S2 The compiled prey isotope values from the literature and unpublished data used in Figure 2.
(DOC)

File S3 The ontogeny of δ^13^C values for the 15 white sharks analyzed for this study. Individuals are in the same A, B, and C groups as Figure 1.
(TIF)

## References

[1] Ford JKB, Ellis GM, Barrett-Lennard LG, Morton AB, Palm RS, et al (1998) Dietary specialization in two sympatric populations of killer whales (*Orcinus orca*) in coastal British Columbia and adjacent waters. Can J Zool 76: 1456–1471.

[2] Bolnick DI, Svanbäck R, Fordyce JA, Yang LH, Davis JM, et al (2003) The ecology of individuals: incidence and implications of individual specialization. Am Nat 161: 1–28 doi:10.1086/343878. 12650459 10.1086/343878

[3] Tinker M, Costa D, Estes J, Wieringa N (2007) Individual dietary specialization and dive behaviour in the California sea otter: using archival time–depth data to detect alternative foraging strategies. Deep Sea Research Part II: Topical Studies in Oceanography 54: 330–342 doi:10.1016/j.dsr2.2006.11.012.

[4] Tinker MT, Bentall G, Estes JA (2008) Food limitation leads to behavioral diversification and dietary specialization in sea otters. Proceedings of the National Academy of Sciences 105: 560. 10.1073/pnas.0709263105 PMC2206575 18195370

[5] Quevedo M, Svanbäck R, Eklöv P (2009) Intrapopulation niche partitioning in a generalist predator limits food web connectivity. Ecology 90: 2263–2274. 19739388 10.1890/07-1580.1

[6] Myers RA, Baum JK, Shepherd TD, Powers SP, Peterson CH (2007) Cascading effects of the loss of apex predatory sharks from a coastal ocean. Science 315: 1846–1850 doi:10.1126/science.1138657. 17395829 10.1126/science.1138657

[7] Baum JK, Worm B (2009) Cascading top-down effects of changing oceanic predator abundances. J Anim Ecology 78: 699–714 doi:10.1111/j.1365–2656.2009.01531.x. 10.1111/j.1365-2656.2009.01531.x 19298616

[8] LeBouef BJ, Reidman M, Keyes RS (1982) White shark predation on pinnipeds in California coastal waters. Fishery Bulletin: 891–895.

[9] Klimley A (1985) The areal distribution and autoecology of the white shark, *Carcharodon carcharias*, off the west coast of North America. In: Sibley G, editor. Biology of the White Shark: A Symposium. Los Angeles: Southern California Academy of Sciences, Vol. 9: 15–40.

[10] Klimley AP, Anderson SD, Pyle P, Henderson R (1992) Spatiotemporal patterns of white shark (*Carcharodon carcharias*) predation at the South Farallon Islands, California. Copeia: 680–690.

[11] Long DJ, Jones RE (1996) White shark predation and scavenging on cetaceans in the eastern North Pacific. In: Klimley AP, Ainley DG, editors. Great White Sharks: The Biology of *Carcharodon carcharias*. San Diego: Academic Press. 293–307.

[12] Long DJ, Hanni KD, Pyle P, Roletto J, Jones RE, et al (1996) White shark predation on four pinniped species in central california waters: geographic and temporal patterns inferred from wounded carcasses. In: Klimley AP, Ainley DP, editors. Great White Sharks: The Biology of *Carcharodon carcharias*. San Diego: Academic Press. 309–316.

[13] Brown AC, Lee DE, Bradley RW, Anderson S (2010) Dynamics of white shark predation on pinnipeds in california: effects of prey abundance. Copeia 2010: 232–238 doi:10.1643/CE-08-012.

[14] Boustany AM, Davis SF, Pyle P, Anderson SD, Le Boeuf BJ, et al (2002) Satellite tagging: expanded niche for white sharks. Nature 415: 35–36. 10.1038/415035b 11780105

[15] Weng KC, Boustany AM, Pyle P, Anderson SD, Brown A, et al (2007) Migration and habitat of white sharks (*Carcharodon carcharias*) in the eastern Pacific Ocean. Mar Biol 152: 877–894 doi:10.1007/s00227-007-0739-4.

[16] Jorgensen SJ, Reeb CA, Chapple TK, Anderson S, Perle C, et al (2010) Philopatry and migration of Pacific white sharks. Proceedings of the Royal Society B: Biological Sciences 277: 679–688 doi:10.1098/rspb.2009.1155. 19889703 10.1098/rspb.2009.1155 PMC2842735

[17] Domeier M, Nasby-Lucas N (2008) Migration patterns of white sharks *Carcharodon carcharias* tagged at Guadalupe Island, Mexico, and identification of an eastern Pacific shared offshore foraging area. Mar Ecol Prog Ser 370: 221–237 doi:10.3354/meps07628.

[18] Carlisle AB, Kim SL, Semmens BX, Madigan DJ, Jorgensen SJ, et al (2012) Using Stable Isotope Analysis to Understand the Migration and Trophic Ecology of Northeastern Pacific White Sharks (*Carcharodon carcharias*). PLoS ONE 7: e30492 doi:10.1371/journal.pone.0030492.t006. 22355313 10.1371/journal.pone.0030492 PMC3280240

[19] Compagno L (2001) Sharks of the World, vol. 2. Food and Agriculture Organization of the United Nations, Rome, Italy. pp.

[20] Deniro MJ, Epstein S (1978) Influence of Diet on Distribution of Carbon Isotopes in Animals. Geochimica et Cosmochimica Acta 42: 495–506.

[21] Goericke R, Fry B (1994) Variations of marine plankton δ^13^C with latitude, temperature, and dissolved CO_2_ in the world ocean. Global Biogeochem Cycles 8: 85–90.

[22] Koch PL (2007) Isotopic study of the biology of modern and fossil vertebrates. In: Michener R, Lajtha K, editors. Stable Isotopes in Ecology and Environmental Science. Malden: Blackwell Publishing. 99–154.

[23] Graham BS, Koch PL, Newsome SD, McMahon KW, Aurioles D (2010) Using isoscapes to trace the movements and foraging behavior of top predators in oceanic ecosystems. Isoscapes: 299–318.

[24] Dugdale R, Goering J (1967) Uptake of new and regenerated forms of nitrogen in primary productivity. Limnology and Oceanography: 196–206.

[25] Saino T, Hattori A (1987) Geographical variation of the water column distrubution of suspended particulate organic nitrogen and its ^15^N natural abundance in the Pacific and its marginal seas. Deep Sea Research Part A Oceanographic Research Papers 34: 807–827.

[26] Altabet MA, Francois R (1994) Sedimentary Nitrogen Isotopic Ratio as a Recorder for Surface Ocean Nitrate Utilization. Global Biogeochem Cycles 8: 103–116.

[27] Voss M, Dippner JW, Montoya JP (2001) Nitrogen isotope patterns in the oxygen-deficient waters of the Eastern Tropical North Pacific Ocean. Deep-Sea Research Part I-Oceanographic Research Papers 48: 1905–1921.

[28] Burton RK, Koch PL (1999) Isotopic tracking of foraging and long-distance migration in northeastern Pacific pinnipeds. Oecologia 119: 578–585. 28307717 10.1007/s004420050822

[29] Newsome SD, Clementz MT, Koch PL (2010) Using stable isotope biogeochemistry to study marine mammal ecology. Marine Mammal Science 26: 509–572 doi:10.1111/j.1748–7692.2009.00354.x.

[30] Reich KJ, Bjorndal KA, Martínez del Rio C (2008) Effects of growth and tissue type on the kinetics of ^13^C and ^15^N incorporation in a rapidly growing ectotherm. Oecologia 155: 651–663 doi:10.1007/s00442–007–0949-y. 18188602 10.1007/s00442-007-0949-y

[31] McClellan CM, Braun-McNeill J, Avens L, Wallace BP, Read AJ (2010) Stable isotopes confirm a foraging dichotomy in juvenile loggerhead sea turtles. Journal of Experimental Marine Biology and Ecology 387: 44–51.

[32] Vander Zanden HB, Bjorndal KA, Reich KJ, Bolten AB (2010) Individual specialists in a generalist population: results from a long-term stable isotope series. Biology Letters 6: 711–714. 20335202 10.1098/rsbl.2010.0124 PMC2936143

[33] Matich P, Heithaus MR, Layman CA (2010) Contrasting patterns of individual specialization and trophic coupling in two marine apex predators. J Anim Ecology 80: 294–305 doi:10.1111/j.1365-2656.2010.01753.x. 10.1111/j.1365-2656.2010.01753.x 20831730

[34] Fisk AT, Tittlemier SA, Pranschke JL, Norstrom RJ (2002) Using anthropogenic contaminants and stable isotopes to assess the feeding ecology of greenland sharks. Ecology 83: 2162–2172.

[35] Estrada J, Rice A, Lutcavage M, Skomall G (2003) Predicting trophic position in sharks of the north-west Atlantic Ocean using stable isotope analysis. J Mar Biol Assoc Uk 83: 1347–1350.

[36] Estrada JA, Rice AN, Natanson LJ, Skomal GB (2006) Use of isotopic analysis of vertebrae in reconstructing ontogenetic feeding ecology in white sharks. Ecology 87: 829–834. 16676526 10.1890/0012-9658(2006)87[829:uoiaov]2.0.co;2

[37] Domi N, Bouquegneau JM, Das K (2005) Feeding ecology of five commercial shark species of the Celtic Sea through stable isotope and trace metal analysis. Marine Environmental Research 60: 551–569. 15925404 10.1016/j.marenvres.2005.03.001

[38] MacNeil MA, Skomal GB, Fisk AT (2005) Stable isotopes from multiple tissues reveal diet switching in sharks. Mar Ecol Prog Ser 302: 119–206.

[39] Papastamatiou YP, Friedlander AM, Caselle JE, Lowe CG (2010) Long-term movement patterns and trophic ecology of blacktip reef sharks (*Carcharhinus melanopterus*) at Palmyra Atoll. Journal of Experimental Marine Biology and Ecology 386: 94–102.

[40] Hutchinson G (1992) Population studies: animal ecology and demography. Bulletin of Mathematical Biology.

[41] Newsome SD, Martínez del Rio C, Bearhop S, Phillips DL (2007) A niche for isotopic ecology. Front Ecol Environ 5: 429–436 doi:10.1890/060150.01.

[42] Bearhop S, Adams CE, Waldron S, Fuller RA, Macleod H (2004) Determining trophic niche width: a novel approach using stable isotope analysis. J Anim Ecology 73: 1007–1012.

[43] Newsome SD, Tinker MT, Monson DH, Oftedal OT, Ralls K, et al (2009) Using stable isotopes to investigate individual diet specialization in California sea otters (*Enhydra lutris nereis*). Ecology 90: 961–974. 19449691 10.1890/07-1812.1

[44] Koch PL, Fisher DC, Dettman D (1989) Oxygen isotope variation in the tusks of extinct proboscideans - a measure of season of death and seasonality. Geology 17: 515–519.

[45] Hobson KA, Schell DM (1998) Stable carbon and nitrogen isotope patterns in baleen from eastern Arctic bowhead whales (*Balaena mysticetus*). Can J Fish Aquat Sci 55: 2601–2607.

[46] Kerr LA, Andrews AH, Cailliet GM, Brown TA, Coale KH (2006) Investigations of Δ^14^C, δ^13^C, and δ^15^N in vertebrae of white shark (*Carcharodon carcharias*) from the eastern North Pacific Ocean. Environ Biol Fish 77: 337–353 doi:10.1007/s10641-006-9125-1.

[47] Kelly B, Dempson JB, Power M (2006) The effects of preservation on fish tissue stable isotope signatures. J Fish Biology 69: 1595–1611.

[48] Kim SL, Koch PL (2012) Methods to collect, preserve, and prepare elasmobranch tissues for stable isotope analysis. Environ Biol Fish 95: 53–63 doi:10.1007/s10641-011-9860-9.

[49] Natanson LJ, Mello JJ, Campana SE (2002) Validated age and growth of the porbeagle shark (*Lamna nasus*) in the western North Atlantic Ocean. Fishery Bulletin 100: 266–278.

[50] Long ES, Sweitzer RA, Diefenbach DR, Ben-David M (2005) Controlling for anthropogenically induced atmospheric variation in stable carbon isotope studies. Oecologia 146: 148–156 doi:10.1007/s00442-005-0181-6. 16082561 10.1007/s00442-005-0181-6

[51] Francey R, Allison C, Etheridge D, Trudinger C, Enting I, et al (1999) A 1000-year high precision record of δ^13^C in atmospheric CO_2_ . Tellus B 51: 170–193.

[52] Ainley DG, Henderson AC, Huber HP, Boekelheide RJ, Allen SG, et al (1985) Dynamics of white shark/pinniped interactions in the Gulf of the Farallones. In: Sibley G, editor. Biology of the White Shark: A Symposium. Los Angeles: Southern California Academy of Sciences, Vol. 9: 109–122.

[53] Hubbell G (1996) Using tooth structure to determine the evolutionary history of the white shark. In: Klimley AP, Ainley DG, editors. Great White Sharks: The Biology of *Carcharodon carcharias*. San Diego: Academic Press. 9–18.

[54] Bertalanffy von L (1938) A quantitative theory of organic growth (inquiries on growth laws II. Human Biol 10: 181–213.

[55] Cailliet GM, Natanson L, Welden B, Ebert D (1985) Preliminary studies on the age and growth of the white shark, *Carcharodon carcharias*, using vertebral bands. In: Sibley G, editor. Biology of the White Shark: A Symposium. Los Angeles: Southern California Academy of Sciences, Vol. 9: 49–60.

[56] Wintner SP, Cliff G (1999) Age and growth determination of the white shark, *Carcharodon carcharias*, from the east coast of South Africa. Fishery Bulletin 97: 153–169.

[57] Moore JW, Semmens BX (2008) Incorporating uncertainty and prior information into stable isotope mixing models. Ecology Letters 11: 470–480 doi:10.1111/j.1461-0248.2008.01163.x. 18294213 10.1111/j.1461-0248.2008.01163.x

[58] Newsome SD, Koch PL, Etnier MA, Aurioles-Gambao D (2006) Using carbon and nitrogen isotope values to investigate maternal strategies in northeast Pacific otariids. Marine Mammal Science 22: 556–572.

[59] Toperoff AK (2002) Examination of diet of harbor porpoise (*Phocoena phocoena*) from central California using stomach content and stable isotope analysis from multiple tissues. San Jose. p.

[60] Graham BS (2008) Trophic dynamics and movements of tuna in the tropical Pacific Ocean inferred from stable isotope analyses. University of Hawai'i at Manoa. p.

[61] Sydeman WJ, Hobson KA, Pyle P, McLaren EB (1997) Trophic relationships among seabirds in central California: combined stable isotope and conventional dietary approach. Condor 99: 327–336.

[62] Kim SL, Casper DR, Galván-Magaña F, Ochoa-Díaz R, Hernández-Aguilar SB, et al (2012) Carbon and nitrogen discrimination factors for elasmobranch soft tissues based on a long-term controlled feeding study. Environ Biol Fish 95: 37–52 doi:10.1007/s10641-011-9919-7.

[63] Gould P, Ostrom P, Walker W (1997) Trophic relationships of albatrosses associated with squid and large-mesh drift-net fisheries in the North Pacific Ocean. Can J Zool 75: 549–562.

[64] Ochoa Díaz R (2009) Espectro trófico del tiburón martillo *Sphyrna zygaena* (Linnaeus, 1758 ) en Baja California Sur: aplicación de ^13^C y ^15^N. La Paz: Centro Interdisciplinario de Ciencias Marinas. p.

[65] Pianka ER (1974) Niche overlap and diffuse competition. Proceedings of the National Academy of Sciences 71: 2141. 10.1073/pnas.71.5.2141 PMC388403 4525324

[66] Lu R-P, Smith EP, Good IJ (1989) Multivariate measures of similarity and niche overlap. Theor Popul Biol 35: 1–21. 2711352 10.1016/0040-5809(89)90007-5

[67] Sydeman WJ, Allen SG (1999) Pinniped population dynamics in central California: Correlations with sea surface temperature and upwelling indices. Marine Mammal Science 15: 446–461.

[68] Carretta JV, Forney KA, Muto MM, Barlow J, Baker J, et al. (2007) U.S. Pacific Marine Mammal Stock Assessments: 2006 National Coean and Atmospheric Administration. p.321.

[69] Anderson DR, Burnham KP, White GC (1998) Comparison of Akaike information criterion and consistent Akaike information criterion for model selection and statistical inference from capture-recapture studies. Journal of Applied Statistics 25: 263–282.

[70] Deniro MJ, Epstein S (1981) Influence of diet on the distribution of nitrogen isotopes in animals. Geochimica et Cosmochimica Acta 45: 341–351.

[71] Tieszen LL, Boutton TW, Tesdahl K, Slade NA (1983) Fractionation and turnover of stable carbon isotopes in animal tissues: implications for δ^13^C analysis of diet. Oecologia 57: 32–37. 28310153 10.1007/BF00379558

[72] Post DM (2002) The long and short of food-chain length. Trends in Ecology & Evolution 17: 269–277.

[73] Gannes LZ, Obrien DM, Martínez del Rio C (1997) Stable isotopes in animal ecology: assumptions, caveats, and a call for more laboratory experiments. Ecology 78: 1271–1276.

[74] Martínez del Rio C, Wolf N, Carleton SA, Gannes LZ (2009) Isotopic ecology ten years after a call for more laboratory experiments. Biological Reviews 84: 91–111 doi:10.1111/j.1469-185X.2008.00064.x. 19046398 10.1111/j.1469-185X.2008.00064.x

[75] Ishiyama R (1951) Studies on the rays and skates belonging to the family Rajidae, found in Japan and adjacent regions. 2. On the age-determination of the Japanese black-skate Raja fusca Garman (Preliminary report). 3. Age determination of Raja hollandi Jordan et Richardson chiefly inhabiting waters of the east China Sea. Bull Japanese Soc Sci Fish 16: 112.

[76] Ainsley SM (2009) Age, growth and reproduction of the Bering skate, *Bathyraja interrupta* (Gill & Townsend, 1897), from the eastern Bering Sea and Gulf of Alaska.

[77] Kim SL, Martínez del Rio C, Casper D, Koch PL (2012) Isotopic incorporation rates for shark tissues from a long-term captive feeding study. Journal of Experimental Biology 215: 2495–2500 doi:10.1242/jeb.070656. 22723489 10.1242/jeb.070656

[78] Ruiz-Cooley RI, Markaida U, Gendron D, Aguinga S (2006) Stable isotopes in jumbo squid (*Dosidicus gigas*) beaks to estimate its trophic position: comparison between stomach contents and stable isotopes. J Mar Biol Assoc Uk 86: 437–445.

[79] Cailliet GM, Smith WD, Mollet HF, Goldman KJ (2006) Age and growth studies of chondrichthyan fishes: the need for consistency in terminology, verification, validation, and growth function fitting. Environ Biol Fish 77: 211–228 doi:10.1007/s10641-006-9105-5.

[80] Kranenbarg S (2005) Adaptive bone formation in acellular vertebrae of sea bass (*Dicentrarchus labrax L.*). Journal of Experimental Biology 208: 3493–3502 doi:10.1242/jeb.01808. 16155222 10.1242/jeb.01808

[81] Deschamps MH, Girondot M, Labbe L, Sire JY (2009) Changes in vertebral structure during growth of reared rainbow trout, Oncorhynchus mykiss (Walbaum): a new approach using modelling of vertebral bone profiles. Journal of Fish Diseases 32: 233–246. 19309418 10.1111/j.1365-2761.2008.00979.x

[82] Bocherens H, Drucker D (2003) Trophic level isotopic enrichment of carbon and nitrogen in bone collagen: case studies from recent and ancient terrestrial ecosystems. Int J Osteoarchaeol 13: 46–53 doi:10.1002/oa.662.

[83] Fox-Dobbs K, Bump JK, Peterson RO, Fox DL, Koch PL (2007) Carnivore-specific stable isotope variables and variation in the foraging ecology of modern and ancient wolf populations: case studies from Isle Royale, Minnesota, and La Brea. Can J Zool 85: 458–471 doi:10.1139/Z07–018.

[84] Jim S, Jones V, Ambrose SH, Evershed RP (2006) Quantifying dietary macronutrient sources of carbon for bone collagen biosynthesis using natural abundance stable carbon isotope analysis. Brit J Nutr 95: 1055–1062. 16768826 10.1079/bjn20051685

[85] Hussey NE, Brush J, McCarthy ID, Fisk AT (2009) δ^15^N and δ^13^C diet–tissue discrimination factors for large sharks under semi-controlled conditions. Comparative Biochemistry and Physiology, Part A: 1–9. doi:10.1016/j.cbpa.2009.09.023. 10.1016/j.cbpa.2009.09.023 19800980

[86] Malpica-Cruz L, Herzka SZ, Sosa-Nishizaki O, Lazo JP, Trudel M (2012) Tissue-specific isotope trophic discrimination factors and turnover rates in a marine elasmobranch: empirical and modeling results. Can J Fish Aquat Sci 69: 551–564 doi:10.1139/f2011–172.

[87] Robbins CT, Felicetti LA, Florin ST (2009) The impact of protein quality on stable nitrogen isotope ratio discrimination and assimilated diet estimation. Oecologia 162: 571–579 doi:10.1007/s00442-009-1485-8. 19898979 10.1007/s00442-009-1485-8

[88] Caut S, Angulo E, Courchamp F (2009) Variation in discrimination factors (Δ^15^N and Δ^13^C): the effect of diet isotopic values and applications for diet reconstruction. Journal of Applied Ecology 46: 443–453 doi:10.1111/j.1365-2664.2009.01620.x.

[89] Howland MR, Corr LT, Young SMM, Jones V, Jim S, et al (2003) Expression of the dietary isotope signal in the compound-specific delta(13) values of pig bone lipids and amino acids. Int J Osteoarchaeol 13: 54–65.

[90] Jim S, Ambrose SH, Evershed RP (2004) Stable carbon isotopic evidence for differences in the dietary origin of bone cholesterol, collagen and apatite: Implications for their use in palaeodietary reconstruction. Geochimica et Cosmochimica Acta 68: 61–72.

[91] Van Valen L (1965) Morphological variation and width of ecological niche. Amer Natur 99: 377–390.

[92] Estes J, Riedman M, Staedler M, Tinker M, Lyon B (2003) Individual variation in prey selection by sea otters: patterns, causes and implications. J Anim Ecology 72: 144–155.

[93] Woo KJ, Elliott KH, Davidson M, Gaston AJ, Davoren GK (2008) Individual specialization in diet by a generalist marine predator reflects specialization in foraging behaviour. J Anim Ecology 77: 1082–1091. 10.1111/j.1365-2656.2008.01429.x 18624834

