# Supplementary figures and images for: Ontogenetic and Among-Individual Variation in Foraging Strategies of Northeast Pacific White Sharks Based on Stable Isotope Analysis
Source: PLoS One. 2012 Sep 28;7(9):e45068. doi: 10.1371/journal.pone.0045068 (PMC3460992; doi:10.1371/journal.pone.0045068)

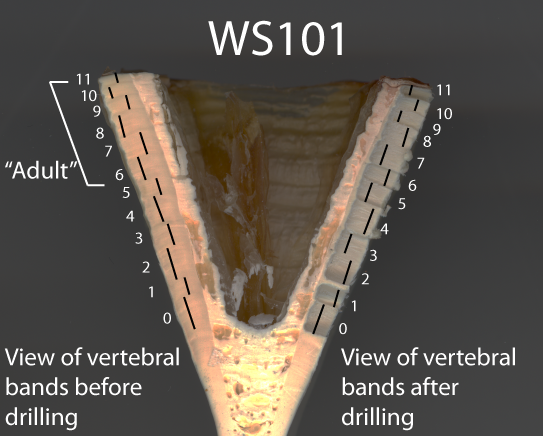

Supplement: File S1 — Photo showing annual growth bands before (left) and after drilling (right) on specimen WS101. Ages are also noted next to the growth bands. (TIF) [file pone.0045068.s001.tif]

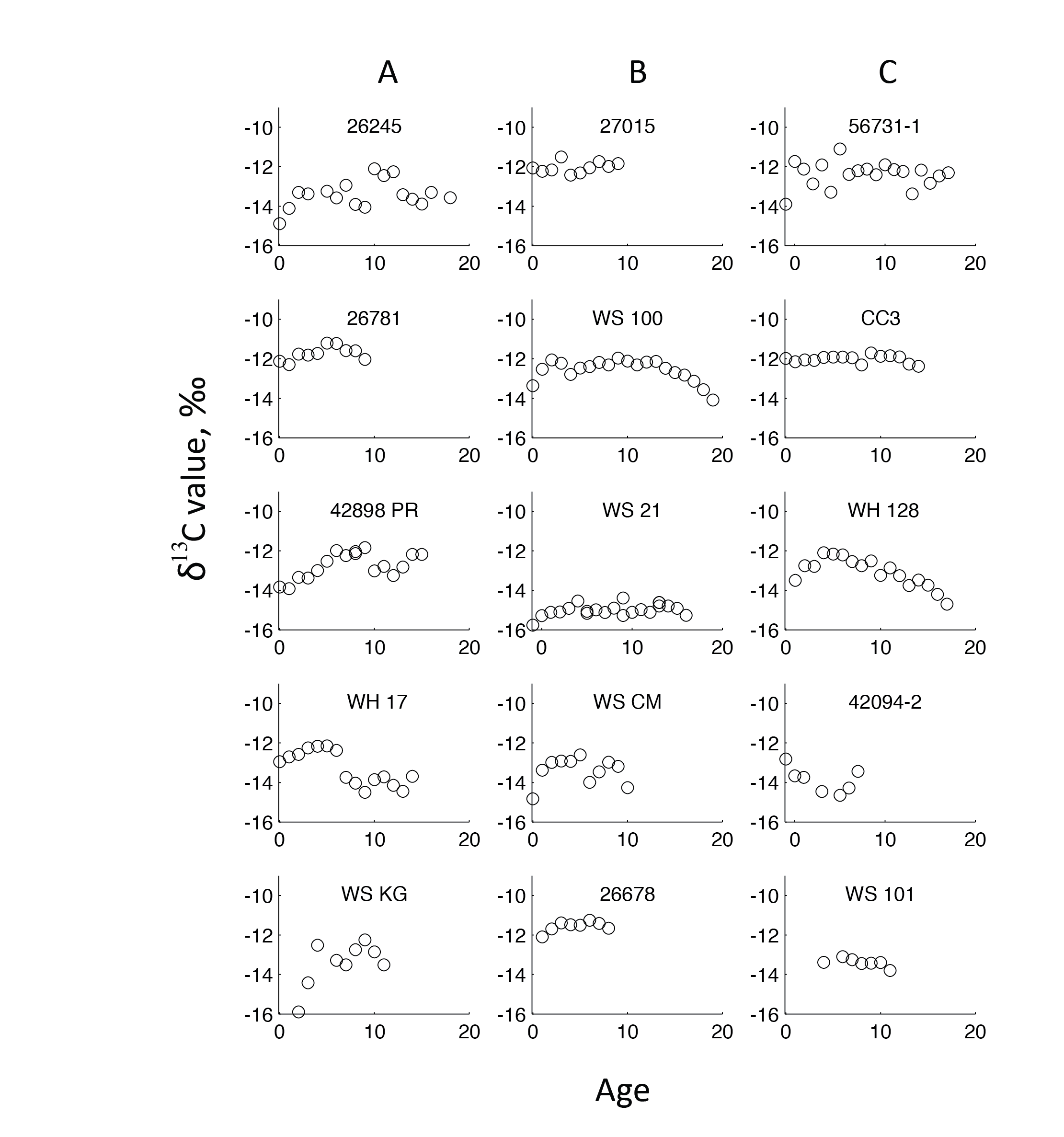

Supplement: File S3 — The ontogeny of δ13C values for the 15 white sharks analyzed for this study. Individuals are in the same A, B, and C groups as Figure 1. (TIF) [file pone.0045068.s003.tif]
