# Supplementary material for: Ontogenetic and Among-Individual Variation in Foraging Strategies of Northeast Pacific White Sharks Based on Stable Isotope Analysis
Source: PLoS One. 2012 Sep 28;7(9):e45068. doi: 10.1371/journal.pone.0045068 (PMC3460992; doi:10.1371/journal.pone.0045068)
Supplement: File S2 — The compiled prey isotope values from the literature and unpublished data used in Figure 2. (DOC) [file pone.0045068.s002.doc]

All data are from collagen, muscle, and keratin samples, which are protein-based substrates. The only published study comparing marine mammal muscle to collagen found collagen to be ~2‰ enriched in ^13^C relative to muscle [1]. An unpublished study by Toperoff [2] compared collagen and muscle isotope values from individual wild porpoises and found significant differences. Bone was consistently ^13^C-enriched relative to muscle (1.5 - 3.9‰), but the δ^15^N offset (bone-muscle) was highly variable (nitrogen: -1.6 - 1.6‰). An often-cited study by Sholto-Douglas et al. [3] compares collagen and muscle isotope values, but these are for fish rather than mammals, which have different metabolic physiologies. We chose to “correct” collagen to muscle values for carbon only based on these previous studies as follows: δ^13^C_collagen_ – 2‰ = δ^13^C_muscle_. Several studies have demonstrated significant offsets between squid beak and muscle, but the values differ substantially [4-6]so we did not correct nearshore cephalopod beaks to muscle. The prey values listed in this appendix are the raw values reported in the original studies.

Prey data were not corrected for the Suess effect due to the recent dates of collection and large variability among individual specimens. Values plotted in Figure 2 are these raw data (shown in bold) corrected for trophic discrimination (4.2‰ and 2.5‰ for δ^13^C and δ^15^N values, respectively). [2,4,7-12]

| Common name (number in Figure 2) | Scientific name | Tissue | Mean δ^13^C (SD), ‰ | Mean δ^15^N (SD), ‰ | locality | Citation |
| --- | --- | --- | --- | --- | --- | --- |
| ***Marine mammals*** | | | | | | |
| **Northern elephant seal (1)** | *Mirounga angustirostris* | collagen | **-13.6 (1.1)** | **18.2 (0.7)** | California | [7] |
| California sea lion | *Zalophus californianus* | collagen | -12.7 (0.6) | 18.4 (0.8) | Pacific Baja California, Mexico | [8] |
| California sea lion | *Zalophus californianus* | collagen | -13.8 (0.9) | 18.5 (0.9) | Southern California | [8] |
| California sea lion | *Zalophus californianus* | collagen | -13.8 (1.1) | 18.6 (0.9) | California | [8] |
| **California sea lion (2)** | |  | **-13.4 (1.5)** | **18.5 (1.5)** |  |  |
| **Harbor seal (3)** | *Phoca vitulina* | collagen | **-12.4 (0.6)** | **18.7 (1.0)** | California | [7] |
| Short-beaked common dolphin | *Delphinus delphis* | collagen | -13.6 (0.6) | 17.2 (1.2) | NE Pacific | Berman and Newsome, unpublished data |
| Long-beaked common dolphin | *Delphinus capensis* | collagen | -13.0 (0.5) | 16.8 (1.0) | NE Pacific | Berman and Newsome, unpublished data |
| **Dolphins (4)** | |  | **-13.3 (0.8)** | **17.0 (1.6)** | NE Pacific |  |
| Harbor porpoise (5) | *Phocena phocena* | muscle | **-16.2 (0.4)** | **15.2 (0.7)** | California | [2] |
| ***Fish*** | | | | | | |
| yellowfin tuna | *Thunnus albacares* | muscle | -15.8 (0.5) | 13 (2.7) | N. central Pacific | [9] |
| skipjack tuna | *Katsuwonus pelamis* | muscle | -15.6 (0.5) | 12.8 (2.6) | N. central Pacific | [9] |
| bigeye tuna | *Thunnus obesus* | muscle | -15.8 (0.6) | 14.5 (2.9) | N. central Pacific | [9] |
| **Tuna (6)** | |  | **-15.7 (0.9)** | **13.4 (4.7)** | N. central pacific |  |
| Anchovy | *Engraulis mordax* | muscle | -16.8 (0.4) | 13.9 (0.8) | California | [10] |
| King salmon | *Oncorhyncus tshawytscha* | muscle | -17.5 (0.2) | 13.8 (0.2) | California | [10] |
| Lingcod | *Ophiodon elongatus* | muscle | -18.3 (0.5) | 12.5 (0.7) | California | [10] |
| Sablefish | *Anoplopoma fimbria* | muscle | -17.3 (0.2) | 12.8 (0.7) | California | [10] |
| Jack mackerel | *Trachurus symmetricus* | muscle | -17.2 (0.5) | 15.0 (0.1) | California | [10] |
| Midship-men | *Porichthys notatus* | muscle | -17.1 (0.6) | 13.9 (0.4) | California | [10] |
| Anchovy | *Engraulis mordax* | muscle | -16.9 (0.8) | 13.7 (0.9) | California | [10] |
| Sardine | *Sardinops sagax* | muscle | -17.7 (0.5) | 13.2 (0.4) | California | [10] |
| Surf smelt | *Hypomesus pretiosus* | muscle | -18.6 (0.6) | 13.4 (0.1) | California | [10] |
| Rockfish | *Sebastes spp.* | muscle | -16.9 (0.1) | 13.3 (0.3) | California | [10] |
| Jack mackerel | *Trachurus symmetricus* | muscle | -17.2 (0.5) | 15.0 (0.1) | California | [10] |
| **Nearshore fish (7)** | |  | **-17.4 (1.6)** | **13.7 (1.7)** |  |  |
| ***Sharks*** | | | | | | |
| Blue sharks (10) | *Prionace glauca* | muscle | **-17.3 (0.6)** | **17.8 (0.6)** | Pacific Baja California, Mexico | [4] |
| Hammer- head sharks (11) | *Sphyrna zygaena* | muscle | **-16.7 (0.4)** | **18.9 (0.8)** | Pacific Baja California, Mexico | [4] |
| ***Cephalopods*** | | | | | | |
| squid | *Eucleoteuthis luminosa* | muscle | -17.8 (0.3) | 11.2 (0.2) | North central Pacific | [11] |
| squid | *Ommastrephes bartrami* | muscle | -18.4 (0.2) | 11.7 (0.4) | North central Pacific | [11] |
| squid | *Berryteuthis anonychus, Octopoteuthis* | muscle | -18.5 (0.4) | 11.6 (1.9) | North central Pacific | [11] |
| squid (neon flying) | *Ommastrephes bartrami* | muscle | -18.4 (0.2) | 11.7 (0.4) | North central Pacific | [11] |
| squid (miscellaneous) | *Berryteuthis anonychus, Octopoteuthis deletron, Histioteuthis dofleini, Taonius pavo* | muscle | -18.5 (0.4) | 11.6 (1.9) | North central Pacific | [11] |
| **Offshore cephalopods (8)** | |  | **-18.3 (0.4)** | **11.6 (0.6)** | North central Pacific |  |
| humbodlt squid | *Dosidicus gigas* | beak | -17.9 | 15.6 | Pacific Baja California, Mexico | [12] |
| humbodlt squid | *Dosidicus gigas* | beak | -16.2 | 15.3 | Pacific Baja California, Mexico | [12] |
| humbodlt squid | *Dosidicus gigas* | beak | -16.7 | 13.5 | Pacific Baja California, Mexico | [12] |
| Octopus | *Octopodotheuthis sp.* | beak | -18.9 | 14.8 | Pacific Baja California, Mexico | [12] |
| hooked squid | *Onychoteuthis banksii* | beak | -17.8 | 13.3 | Pacific Baja California, Mexico | [12] |
| diamond back squid | *Thysanoteuthis rhombus* | beak | -18.7 | 12.1 | Pacific Baja California, Mexico | [12] |
| flying squid | *Stenotheuthis oualaniensis* | beak | -17.9 | 12.8 | Pacific Baja California, Mexico | [12] |
| Sharpear Enope squid | *Ancistrocheirus lesueurii* | beak | -16.1 | 15.1 | Pacific Baja California, Mexico | [12] |
| hooked squid | *Onychoteuthis banksii* | beak | -17.8 | 12.5 | Pacific Baja California, Mexico | [12] |
| **Nearshore cephalopods (9*)*** | |  | **-17.5 (0.8)** | **13.9 (1.1)** |  |  |

1. Ramsay MA, Hobson KA (1991) Polar bears make little use of terrestrial food webs - evidence from stable-carbon isotope analysis. Oecologia 86: 598–600.

2. Toperoff AK (2002) Examination of diet of harbor porpoise (*Phocoena phocoena*) from central California using stomach content and stable isotope analysis from multiple tissues. San Jose. p.

3. Sholto-Douglas A, Field J, James A, Van der Merwe N (1991) ^13^C/^12^C and ^15^N/^14^N isotope ratios in the Southern Benguela Ecosystem: indicators of food web relationships among different size-classes of plankton and pelagic fish; differences between fish muscle and bone collagen tissues. Mar Ecol Prog Ser 78.

4. Kim SL, Casper DR, Galván-Magaña F, Ochoa-Díaz R, Hernández-Aguilar SB, et al. (2012) Carbon and nitrogen discrimination factors for elasmobranch soft tissues based on a long-term controlled feeding study. Environ Biol Fish 95: 37–52. doi:10.1007/s10641-011-9919-7.

5. Ruiz-Cooley RI, Markaida U, Gendron D, Aguinga S (2006) Stable isotopes in jumbo squid (*Dosidicus gigas*) beaks to estimate its trophic position: comparison between stomach contents and stable isotopes. J Mar Biol Assoc Uk 86: 437–445.

6. Hobson KA, Cherel Y (2006) Isotopic reconstruction of marine food webs using cephalopod beaks: new insight from captively raised *Sepia officinalis*. Can J Zool 84: 766–770. doi:10.1139/z06-049.

7. Burton RK, Koch PL (1999) Isotopic tracking of foraging and long-distance migration in northeastern Pacific pinnipeds. Oecologia 119: 578–585.

8. Newsome SD, Koch PL, Etnier MA, Aurioles-Gambao D (2006) Using carbon and nitrogen isotope values to investigate maternal strategies in northeast Pacific otariids. Marine Mammal Science 22: 556–572.

9. Graham BS (2008) Trophic dynamics and movements of tuna in the tropical Pacific Ocean inferred from stable isotope analyses. University of Hawai'i at Manoa. p.

10. Sydeman WJ, Hobson KA, Pyle P, McLaren EB (1997) Trophic relationships among seabirds in central California: combined stable isotope and conventional dietary approach. Condor 99: 327–336.

11. Gould P, Ostrom P, Walker W (1997) Trophic relationships of albatrosses associated with squid and large-mesh drift-net fisheries in the North Pacific Ocean. Can J Zool 75: 549–562.

12. Ochoa Díaz R (2009) Espectro trófico del tiburón martillo *Sphyrna zygaena* (Linnaeus, 1758 ) en Baja California Sur: aplicación de ^13^C y ^15^N. La Paz: Centro Interdisciplinario de Ciencias Marinas. p.
